# A remarkably stable *TipE *gene cluster: evolution of insect Para sodium channel auxiliary subunits

**DOI:** 10.1186/1471-2148-11-337

**Published:** 2011-11-18

**Authors:** Jia Li, Robert M Waterhouse, Evgeny M Zdobnov

**Affiliations:** 1 Department of Genetic Medicine and Development, University of Geneva Medical School, rue Michel-Servet 1, 1211 Geneva, Switzerland; 2 Swiss Institute of Bioinformatics, rue Michel-Servet 1, 1211 Geneva, Switzerland; 3 Imperial College London, South Kensington Campus, London, SW7 2AZ, UK

## Abstract

**Background:**

First identified in fruit flies with temperature-sensitive paralysis phenotypes, the *Drosophila melanogaster TipE *locus encodes four voltage-gated sodium (Na_V_) channel auxiliary subunits. This cluster of *TipE*-like genes on chromosome 3L, and a fifth family member on chromosome 3R, are important for the optional expression and functionality of the Para Na_V _channel but appear quite distinct from auxiliary subunits in vertebrates. Here, we exploited available arthropod genomic resources to trace the origin of *TipE*-like genes by mapping their evolutionary histories and examining their genomic architectures.

**Results:**

We identified a remarkably conserved synteny block of *TipE*-like orthologues with well-maintained local gene arrangements from 21 insect species. Homologues in the water flea, *Daphnia pulex*, suggest an ancestral pancrustacean repertoire of four *TipE*-like genes; a subsequent gene duplication may have generated functional redundancy allowing gene losses in the silk moth and mosquitoes. Intronic nesting of the insect *TipE *gene cluster probably occurred following the divergence from crustaceans, but in the flour beetle and silk moth genomes the clusters apparently escaped from nesting. Across Pancrustacea, *TipE *gene family members have experienced intronic nesting, escape from nesting, retrotransposition, translocation, and gene loss events while generally maintaining their local gene neighbourhoods. *D. melanogaster TipE*-like genes exhibit coordinated spatial and temporal regulation of expression distinct from their host gene but well-correlated with their regulatory target, the Para Na_V _channel, suggesting that functional constraints may preserve the *TipE *gene cluster. We identified homology between TipE-like Na_V _channel regulators and vertebrate Slo-beta auxiliary subunits of big-conductance calcium-activated potassium (BK_Ca_) channels, which suggests that ion channel regulatory partners have evolved distinct lineage-specific characteristics.

**Conclusions:**

*TipE*-like genes form a remarkably conserved genomic cluster across all examined insect genomes. This study reveals likely structural and functional constraints on the genomic evolution of insect *TipE *gene family members maintained in synteny over hundreds of millions of years of evolution. The likely common origin of these Na_V _channel regulators with BK_Ca _auxiliary subunits highlights the evolutionary plasticity of ion channel regulatory mechanisms.

## Background

Voltage-gated sodium (Na_V_) channels control the passage of sodium ions across the plasma membrane and play a crucial role in the creation and propagation of electrical signals in excitable cells, such as neurons and myocytes. The pore-forming alpha subunits comprise a gene family of up to ten members in vertebrate species [1], which are associated with four types of auxiliary beta-subunits that are important for the biogenesis and gating kinetics of functional alpha-subunits [2]. In contrast, only two NaV channel alpha-subunit genes are found in insects, orthologues of *Drosophila melanogaster DSC1 *(*Drosophila sodium channel 1*) and *Para *(*paralytic*), and sequence homology searches fail to identify homologues of the vertebrate Na_V _channel auxiliary beta-subunits. Although DSC1 may rather function as a calcium channel involved in olfactory avoidance responses [1,3,4], *Para *mutants exhibit temperature-sensitive paralysis caused by loss of Na^+^-based action potentials [5-8]. Thus, insect genomes appear to encode only one functional Na_V _channel alpha-subunit gene and no orthologues of the vertebrate Na_V _channel auxiliary beta-subunits.

Studies of temperature-sensitive paralysis phenotypes in *D. melanogaster *identified a role for the *TipE *locus (*temperature-induced paralysis locus E*) in cell excitability, and suggested regulatory interactions with the Para Na_V _channel [9,10]. Subsequent identification of the *TipE *gene facilitated studies that demonstrated its Na_V _channel regulator activity by stimulating functional expression of *Para *in *Xenopus *oocytes, and established that *TipE *expression during pupal development rescued the paralysis phenotype in adult fruit flies [11]. Moreover, sodium-dependent repetitive firing in *Drosophila *neurons is regulated by TipE [12], and Para-TipE interactions influence the pharmacology of the Para Na_V _channel [13]. Despite the lack of insect orthologues of vertebrate Na_V _channel auxiliary beta-subunits, characterization of the *D. melanogaster TipE *gene revealed strikingly similar functions: an auxiliary subunit required for the correct assembly and gating of the Para Na_V _channel. Furthermore, four homologues of *TipE *(*Teh1-4*) are encoded in the *D. melanogaster *genome and their *TipE*-like functions as auxiliary subunits of Para Na_V _channels have been characterized through co-expression with *Para *in *Xenopus *oocytes [14]. Thus, fruit fly *TipE *gene family members are important for the optimal expression and functionality of the Para Na_V _channel but appear quite distinct from auxiliary beta-subunits in vertebrates.

Their key roles in membrane excitability make insect Na_V _channels attractive targets for the development of insecticides [13,15,16]. However, *Para *mutations lead to knockdown resistance in insects and other arthropods, which reduces the efficacy of many insecticides and intensifies the search for alternative targets [17-19]. Characterizing the evolutionary histories and molecular functions of insect *TipE *gene family members will not only further the understanding of the mechanisms controlling cell excitability, but may also offer novel opportunities for developing alternative insect control strategies. In this study, we examined the genealogies of *TipE *gene family members across multiple insect species with sequenced genomes, and revealed a remarkably conserved synteny block of *TipE *homologues with local gene arrangements that have been well-maintained in many insect genomes over hundreds of millions of years of evolution.

## Results and discussion

### A genomic cluster of *TipE *gene family members conserved across Insecta

**Figure 1:**
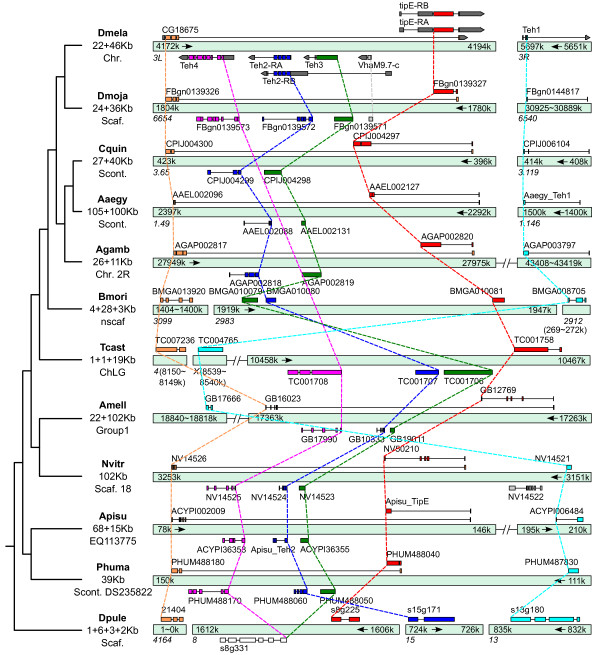
**Genomic organization of *TipE *gene family members in 11 insect species and *Daphnia pulex***. *TipE *gene family members exhibit well-maintained local gene neighbourhoods across Insecta. Orthologues of *Drosophila melanogaster *(Dmela) *TipE *(red), *Teh2 *(blue), and *Teh3 *(green) form a conserved synteny block nested in the last intron of *CG18675 *orthologues (orange), except in the silk moth (*Bombyx mori*, Bmori) and the four beetle (*Tribolium castaneum*, Tcast) where they have escaped from intronic nesting. The synteny block is extended by *Teh4 *orthologues (purple) in all insects except the mosquitoes (*Anopheles gambiae*, Agamb; *Aedes aegypti*, Aaegy; and *Culex quinquefasciatus*, Cquin) and the silk moth. The gene cluster is conserved in fruit flies with the inserted *VhaM9.7-c *gene in Dmela also present in *Drosophila mojavensis *(Dmoja) and ten other drosophilids (not shown). Although *Teh1 *orthologues (cyan) are often not linked to the *TipE *locus, the neighbouring location in the human body louse (*Pediculus humanus*, Phuma) is likely to represent the ancestral arrangement, supported by the genomic proximity of *Teh1 *in the pea aphid (*Acyrthosiphon pisum*, Apisu) and parasitoid wasp (*Nasonia vitripennis*, Nvitr). The honey bee (*Apis mellifera*, Amell) and flour beetle *Teh1 *orthologues have undergone translocation events relative to their *Teh4-Teh2-Teh3-TipE *clusters, but are retained on the same linkage groups. The genome of the crustacean water flea, *Daphnia pulex *(Dpule), encodes orthologues of *TipE, Teh1*, and *Teh2*, but only one *Teh3/4*-like gene (s8g331). As *Teh3 *is a single-coding-exon gene in all the insect genomes, it likely arose from the retrotransposition of a *Teh4*-like gene before the insect radiation. Putative orthologous relations among *TipE *gene family members are indicated with dashed lines. Start and end positions are indicated for scaffolds, supercontigs, linkage groups, or chromosomes with arrows indicating the direction of the forward strand of the reference assemblies. Complete gene models including untranslated regions and alternative transcripts are displayed for Dmela while only coding-sequence regions are depicted for the other species.

High levels of genome shuffling observed in many insect species lead to the rapid decay of synteny, leaving only a few identifiable conserved gene clusters [20]. Examples of such well-maintained local gene arrangements include the *homeobox *(*Hox*) [21] and *wingless *(*Wnt*) [22] gene clusters with key roles in early development. Synteny block analysis across 23 arthropod species with sequenced genomes (see Methods) identified both *Hox *and *Wnt *gene clusters, and highlighted a genomic cluster of *TipE *gene family members as one of the most conserved blocks of orthologues with well-maintained local gene arrangements. This highly conserved synteny block was defined by a core of three sets of orthologous genes from 20 insect species: *Drosophila melanogaster *and 11 other fruit flies; three mosquitoes: *Anopheles gambiae, Aedes aegypti*, and *Culex quinquefasciatus*; the silk moth, *Bombyx mori*; the flour beetle, *Tribolium castaneum*; the parasitoid wasp, *Nasonia vitripennis*; the honey bee, *Apis mellifera*; and the human body louse, *Pediculus humanus*. Sequence homology searches of genomic resources of the remaining sequenced arthropod species revealed a similarly conserved gene arrangement in the pea aphid, *Acyrthosiphon pisum*, as well as four *TipE *homologues in the crustacean water flea, *Daphnia pulex*, but no homologous genes in the arachnid deer tick, *Ixodes scapularis *(possibly due to incomplete genome sequencing). The three *D. melanogaster *genes are located in a ~15 kb region of chromosome 3L and encode the Para Na_V _channel auxiliary subunits, Teh2, Teh3, and TipE. Directly neighbouring the *Teh2 *gene is a fourth homologue, *Teh4*, which, together with *Teh1 *on chromosome 3R, make up the full complement of five *D. melanogaster TipE *gene family members. The conservation of the *Teh2-Teh3-TipE *synteny block across Insecta, despite generally high rates of genomic shuffling [20] and gene losses [23] over more than ~350 million years of evolution [24], suggests that strong constraints have maintained the local genomic architecture of the *TipE *locus (Figure 1).

Comparative analysis of the *TipE *gene family previously identified orthologues of *D. melanogaster TipE, Teh1, Teh2*, and *Teh3 *from *A. gambiae, B. mori*, and *A. mellifera*, as well as orthologues of all five family members from two other fruit flies, *D. pseudoobscura *and *D. yakuba*, and noted their similar genomic clustering [14]. Inspecting the genomic loci of all *TipE *gene family members in *D. pulex *and each of the 21 insect genomes facilitated comprehensive gene model curation to confirm and correct their predicted genomic architectures and protein-coding sequences (see Methods). These analyses identified the previously unannotated *A. pisum TipE *orthologue and the *A. aegypti Teh1 *orthologue, corrected the previously split gene model of the *A. pisum Teh2 *orthologue, and improved several other gene model annotations (see Additional file 1, Table S1 for full details on all corrected gene models). The otherwise strictly-conserved arrangement of the *Teh2-Teh3-TipE *synteny block, with *Teh2 *and *Teh3 *encoded on the opposite strand to *TipE*, is disrupted in the fruit flies by a short gene encoding a Vacuolar H^+ ^ATPase subunit (*VhaM9.7-c *in *D. melanogaster*) between *Teh3 *and *TipE*, and by a relative inversion of *Teh2-Teh3 *in the silk moth (Bmori). *Teh4 *orthologues are located adjacent to *Teh2 *and extend the core synteny block in all the insects except the three mosquitoes and the silk moth, which appear to have lost the *Teh4 *gene. In the genome of the human body louse (Phuma), *Teh1 *is adjacent to *TipE *(~15 kb apart), while in the pea aphid (Apisu) it is ~63 kb away and separated by two intervening genes; in the parasitoid wasp (Nvitr), it is ~24 kb away with only a single intervening gene. As the compact body louse genome is remarkably free from transposable elements and appears to have experienced less shuffling than other insect genomes [25], the gene cluster in the last common ancestor with all five *TipE *gene family members is likely to have resembled the genomic arrangement found in the body louse today.

### Structural constraints: intronic nesting of the *TipE *locus

The evidence-based annotations of the *D. melanogaster TipE *locus provide a comprehensive view of the complex gene architectures and reveal that the coding sequences of this gene cluster - *Teh4, Teh2, Teh3, VhaM9.7-c*, and *TipE *- are all nested within the last intron of the neighbouring *CG18675 *gene (Figure 1). Such nested gene structures in eukaryotes are generally much more frequently gained than lost, suggesting that while intronic insertions may be relatively well-tolerated, deletions are more likely to be constrained due to their possible negative impact on both the nested and the host genes [26]. Single-copy orthologues of *CG18675 *can be identified in almost all sequenced metazoan genomes, as well as in some flagellate protozoa, green plants, and apicomplexan parasites, but appear to be absent from fungi and nematode worms. These gene sequences are relatively well-conserved, with 49% amino acid identity between *CG18675 *and the human orthologue (C21orf59, ENSG00000159079), and are matched by the sequence profile, *domain of unknown function DUF2870 *(IPR021298). Although it has been preserved as a single-copy gene across a broad phyletic range, the nesting of multiple genes within the last intron of *CG18675 *orthologues appears to have only occurred in insects. A genomic sequence gap obscures the start of the water flea (Dpule) *CG18675 *orthologue, nevertheless, the C-terminal coding exons align well with other *CG18675 *orthologues (see Additional file 2), and the last intron contains no nested genes. This suggests that while it is possible that water flea *TipE*-like genes escaped from intronic nesting, it is more likely that the nesting event occurred in the last common ancestor of insects, after the divergence from crustaceans.

The genomic span of insect *CG18675 *orthologues with nested *TipE *gene family members ranges from ~20 kb in the fruit fly (Dmela) and the body louse (Phuma) to ~100 kb in the *Aedes *mosquito (Aaegy), which, in addition to the insertion of the *VhaM9.7-c *gene in the drosophilid lineage, highlights the dynamic evolution of this region. Despite these perturbations, the intronic nesting and local gene arrangements of the *TipE *locus have remained largely intact. In addition to the constraint imposed by intronic nesting, a likely contributing factor is the overlap of the terminal exons of *CG18675 *and *TipE*. These exons contain both coding and untranslated regions, however, their overlapping coding regions are out of phase and encode two distinct terminal peptides of 37 (CG18675) and 20 (TipE) amino acids (see Additional file 3, Figure S1). Nesting of the *TipE *locus and the overlapping terminal exons are observed in the *CG18675 *orthologues from all the examined insect species except the flour beetle (Tcast) and the silk moth (Bmori). The flour beetle *CG18657 *orthologue (TC007236) is on a different linkage group to the *TipE *locus and the silk moth gene (BGIBMGA013920) is on a different scaffold. Both genes show conserved coding sequences that include the last coding exon, indicating that their gene models are complete (see Additional file 2), and their genomic spans of ~2.5 kb (Bmori) and ~1 kb (Tcast) are greatly reduced compared to other *CG18675 *orthologues. Thus, in these representative species from the Coleoptera and Lepidoptera lineages, the *TipE *locus appears to have been released from the constraint of intronic nesting. Despite their escape from intronic nesting, *Teh2-Teh3 *remain adjacent to *TipE *in the silk moth, and in the flour beetle the local gene arrangements of *Teh4, Teh2, Teh3*, and *TipE *are strictly maintained, suggesting possible functional constraints that preserve the architecture of the *TipE *locus.

### Functional constraints: coordinated regulation of *TipE *gene family members

Intronic nesting may suggest possible mechanisms for coordinated regulation of host and nested genes, however, functional relations between host and their nested genes are usually unclear, and their expression profiles often exhibit inverse or no significant correlations [26,27]. Insect *glucose-methanol-choline (GMC) oxidoreductase *genes exhibit a similar nested genomic architecture to that of the *TipE *locus; most gene family members are encoded in a large intron of the *flotillin-2 *(*Flo-2*) gene in *D. melanogaster*, and its orthologues in other insects [20,28]. This cluster of *GMC oxidoreductases *may be coordinately regulated for developmental or physiological roles involved in ecdysteroid metabolism, which may be linked to the function of the host gene product, Flo-2, in lipid rafts [28]. Multiple nesting events of homologous genes within a single host intron could therefore facilitate coordinated regulation of the nested genes. Examining *D. melanogaster *gene expression data from different tissue samples (FlyAtlas [29]) and developmental series experiments (modENCODE [30]) revealed common and distinct spatial and temporal expression patterns of the *TipE *locus genes (see Additional file 4). Tissues with the highest transcript levels of *TipE *gene family members include the brain, thoracicoabdominal ganglion, eye, larval central nervous system, and the head, mirroring the expression of *Para *and consistent with their roles in excitable cells.

The developmental series data reveal two peaks of gene expression; the first, from the 14-16-hour embryo to the first larval stage (L1), is dominated by *Teh4*. The start of the second peak is evident from the 12-hour white prepupae, with maximal transcript levels at the 3-day pupae and maintained expression in adult males, but not in adult females. *TipE-Teh1 *and *Teh2-Teh3 *are the best correlated pairs of expression profiles, *TipE *is also well-correlated with *Teh2 *and *Teh4*, but to a lesser extent with *Teh3 *(Spearman correlation test, Table 1). Thus, coordinated regulation appears not only amongst the intron-nested *TipE *gene family members, but also with the unlinked *Teh1 *gene. However, these coordinated expression profiles exhibit poor correlations with that of the host gene, *CG18657*, which displays a small peak during mid-embryogenesis and then increases again from the 12-hour L3 larval stage with greatly-elevated expression in adult males and almost undetectable levels in adult females, consistent with the high transcript levels observed in testis samples. In contrast, the *TipE *gene family members' expression profiles are much better-matched to that of *Para*, particularly *TipE *and *Teh1 *- these two members also induced the largest increases of expression levels of Para Na_V _channels in *Xenopus *oocytes [14]. Thus, *D. melanogaster TipE *gene family members exhibit coordinated spatial and temporal regulation that also correlates well with the expression of *Para*, their functional Na_V _channel partner, but which appears distinct from the host gene, *CG18657*.

**Table 1:** Spearman correlation coefficient for gene expression data of *D. melanogaster *genes

	*TipE*	*Teh1*	*Teh2*	*Teh3*	*Teh4*	*Para*	*Slowpoke*
***Teh1***	0.94 ***						
***Teh2***	0.82 **	0.79 **					
***Teh3***	0.70 *	0.69 *	0.94 ***				
***Teh4***	0.80 **	0.71 *	0.59 *	0.52 *NS*			
***Para***	0.92 ***	0.90 ***	0.77 **	0.61 *	0.69 *		
***Slowpoke***	0.92 ***	0.92 ***	0.81 *	0.71 *	0.66 *	0.89 ***	
***CG18675***	0.42 *NS*	0.41 *NS*	0.44 *NS*	0.51 *NS*	0.34 *NS*	0.21 *NS*	0.47 *NS*

Spearman correlation coefficient p-values: *** < 1e-09, ** < 1e-06, * < 1e-03, NS not significant

Spearman correlation tests performed on *D. melanogaster *gene expression data from developmental series experiments (see Additional file 4) using R (http://www.r-project.org/) show that the expression profiles of *TipE *gene family members are well-correlated, as well as with *Para *and *Slowpoke*. However, the expression profiles of these genes exhibit poor correlation with the host gene of *TipE *gene cluster, *CG18675*.

### Evolutionary history of the insect *TipE *gene family

**Figure 2:**
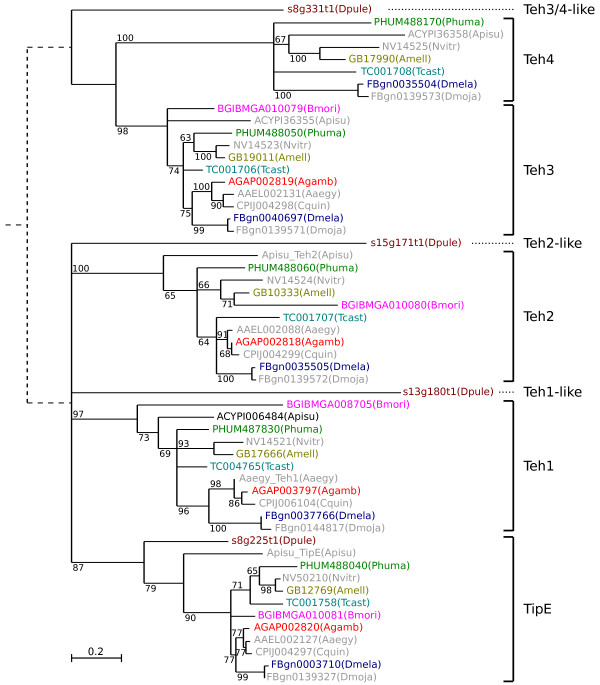
**Phylogenetic tree of *TipE *gene family members in 11 insect species and *Daphnia pulex***. The phylogeny of *TipE *gene family members highlights the five groups of orthologues from the crustacean water flea, *Daphnia pulex *(Dpule) and 11 representative insect species: two fruit flies, *Drosophila melanogaster *(Dmela) and *Drosophila mojavensis *(Dmoja); three mosquitoes, *Anopheles gambiae *(Agamb), *Aedes aegypti *(Aaegy), *Culex quinquefasciatus *(Cquin); the silk moth, *Bombyx mori *(Bmori); the flour beetle, *Tribolium castaneum *(Tcast), the honey bee, *Apis mellifera *(Amell); the parasitoid wasp, *Nasonia vitripennis *(Nvitr); the pea aphid, *Acyrthosiphon pisum *(Apisu); and the human body louse, *Pediculus humanus *(Phuma). The clade of Teh3-Teh4 proteins is distinct from TipE-Teh1-Teh2, linked by dashed lines based on the phylogenetic tree of *TipE *gene family members in *D. pulex *and six selected insect species (coloured), rooted using the distantly related vertebrate big-conductance calcium-activated potassium channel beta-4 auxiliary subunits (KCNMB4) (see Additional file 3, Figure S2). The phylogeny resolves the *D. pulex *TipE orthologue, and although Teh1 and Teh2 orthologues are not confidently resolved, they can be identified by comparing their conserved motif architectures with the insect Teh1 and Teh2 orthologues (see Additional file 3, Figure S3). A single *Teh3/4*-like gene is found in the *D. pulex *genome, which probably reflects the ancestral pancrustacean genome, where a retrotransposition event before the insect radiation generated the *Teh3 *retrogene. This gene duplication may have created a functional redundancy which allowed for the loss of *Teh4 *orthologues in mosquitoes and the silk moth. The maximum likelihood phylogeny was built from the confidently-aligned regions of the multiple sequence alignment of member proteins (see Methods). Bootstrap support values from 100 samples are given and nodes with less than 60% support are collapsed.

Phylogenetic analysis of *TipE *gene family members from *D. pulex *and a representative subset of the 21 insect species highlights the five groups of orthologues (see Figure 2 and Additional file 3, Figure S2). The TipE-Teh1-Teh2 proteins form a distinct clade from Teh3-Teh4, suggesting possible functional divergence between members of these two clades. This is supported by the presence of the epidermal growth factor (EGF)-like domains in the extracellular loop regions of Teh3 and Teh4 proteins that are not found in other *TipE *gene family members, which may interact with extracellular matrix components [14]. Conserved sequence motif analysis using MEME [31] supports the orthology assignments, highlights the extended extracellular loop regions of the Teh3 and Teh4 orthologues, and shows that the sequence regions common to all *TipE *gene family members are comprised of the two transmembrane domains and parts of the extracellular loop (see Additional file 3, Figure S3). These regions exhibit high rates of amino acid substitutions, and this variation may explain the lack of confident bootstrap support for several nodes on the *TipE *gene family phylogeny (see Figure 2 and Additional file 3, Figure S2). The *D. pulex *genome encodes only one *Teh3/4*-like gene and *Teh3 *is found as a single-coding-exon gene in all the examined insect genomes; the alignment of the *D. melanogaster *genes identifies seven sites with uninterrupted coding sequence in *Teh3 *but with introns in *Teh4*, further suggesting that *Teh3 *arose from the retrotransposition of a *Teh4*-like gene of the last common ancestor before the insect radiation. In the silk moth and the three mosquitoes, the absence of *Teh4 *orthologues implies that the *Teh3 *retrogene, which integrated into the same local genomic environment, could have assumed the functions of the original multi-exon ancestor. The *D. pulex *TipE orthologue is resolved on the *TipE *gene family phylogeny (Figure 2), and although Teh1 and Teh2 orthologues are not confidently resolved, they can be identified by comparing their motif architectures (see Additional file 3, Figure S3) with the insect Teh1 and Teh2 orthologues. Thus, it is likely that the ancestral pancrustacean genome encoded four *TipE *gene family members, the subsequent retrotransposition of *Teh4 *after the divergence of the crustacean and insect lineages gave rise to *Teh3 *and created a possible functional redundancy which led to the loss of *Teh4 *in the silk moth and the mosquitoes.

Performing BLAST and PSI-BLAST homology searches of non-redundant sequence databases using *TipE *gene family members failed to identify any significant matches beyond Pancrustacea. Orthologues of *Para *are present in all the examined insects and the water flea, as well as in the closest outgroup species, the arachnid deer tick, *Ixodes scapularis *(see Additional file 1, Table S1 and Additional file 3, Figure S4), however, no *TipE *homologues were identified in either the deer tick's assembled scaffolds or sequence trace archives. Similarly, TipE-like auxiliary beta-subunits are not found in the genome of the nematode worm, *Caenorhabditis elegans*, which also lacks any known Na_V _channel homologues, despite the likely presence of an ancestral sodium channel before the divergence of vertebrates and invertebrates [32]. Scans with Hidden Markov Model (HMM) profiles built from multiple protein sequence alignments of *TipE *gene family members (see Methods) also produced no significant matches to tick or worm proteins. However, the improved search sensitivity of the HMM profiles revealed distant homology with beta-4 auxiliary subunits of vertebrate big-conductance calcium-activated potassium (BK_Ca_) channels (KCNMB4, a member of the Slo-beta or BK-beta subunit family): the HMM profile built from the 32 *TipE *gene family members shown in Additional file 3, Figure S2 identifies human, mouse, cow, and frog KCNMB4 proteins with e-values of less than 1e-05. A reciprocal homology search of arthropod proteomes with a vertebrate KCNMB4 HMM profile identifies insect Teh1 orthologues with e-values of less than 1e-03. A notable difference occurs in the extracellular loop region of TipE, Teh3, and Teh4 proteins, which are longer than the loops in Slo-beta proteins and exhibit EGF-like domains in Teh3 and Teh4 [2,14]. Although the possibility of convergent evolution of these two families of auxiliary subunits cannot be excluded, the similar protein architecture of TipE-like and Slo-beta proteins, with two hydrophobic regions that anchor an extracellular loop containing putative disulphide bridges, further supports their common origin.

Their likely common ancestry with Slo-beta subunits suggests that some *TipE *gene family members may also play a role in the regulation of BK_Ca _channels. This type of ion channel is found across metazoan species, and is named *Slowpoke *in *D. melanogaster*. Although *Slowpoke *exhibits generally higher expression levels than *Para*, particularly in the eye, head, and spermatheca, their developmental expression profiles are well-correlated and match those of the *TipE *gene family members (see Table 1 and Additional file 4). However, rather than suggesting a role for some *TipE *gene family members in the regulation of *Slowpoke*, their similar developmental expression profiles may arise simply because both channels function in excitable cells. Indeed, *TipE *gene family members showed no effects on *Slowpoke *potassium currents in *D. melanogaster *cell lines [14], which are instead regulated by an auxiliary subunit with a protein kinase-like domain, Slowpoke binding protein [33]. The likely common ancestor of the TipE-like and Slo-beta auxiliary subunits may therefore have functioned together with the pore-forming alpha-subunits of an ancestral metazoan potassium or sodium channel, subsequently undergoing independent fine-tuning to control calcium-activated potassium currents in vertebrates and voltage-gated sodium currents in the invertebrate lineage.

## Conclusion

The *TipE *gene family of Para Na_V _channel auxiliary subunits forms a remarkably conserved genomic cluster of orthologues with local gene arrangements maintained across many diverse insect species. This conservation suggests strong constraints that preserve the organisation of the *TipE *gene cluster, as observed for the well-studied *Hox *gene clusters that nevertheless appear to tolerate limited gene rearrangements in *Drosophila *[34]. Comparing the *TipE *synteny block architecture across 21 insect species and examining expression patterns of *D. melanogaster *genes provides evidence of both structural and functional constraints that preserve the *TipE *cluster. The ancestral insect *TipE *gene cluster is likely to have resembled the genomic arrangement found in the human body louse today; the *TipE, Teh2, Teh3*, and *Teh4 *genes nested within the last intron of the *CG18657 *ancestor, and directly neighbouring *Teh1*. Although no *TipE*-like genes were identified in arachnid or nematode genomes, homologues encoded in the *D. pulex *genome suggest an ancient pancrustacean repertoire of four *TipE *gene family members. Retrotransposition of *Teh4 *gave rise to the insect *Teh3 *retrogene following the divergence of crustaceans and insects, which may have generated functional redundancy that allowed for the loss of *Teh4 *in the silk moth and the mosquitoes. Local gene arrangements of the *TipE *locus are well-maintained despite the apparent escape from intronic nesting in the silk moth and the flour beetle genomes, suggesting additional constraints that preserve the genomic architecture. *D. melanogaster *gene expression data reveal coordinated spatial and temporal regulation of the *TipE *gene family members that is distinct from the host gene, but which correlates well with the expression of their regulatory target, Para. Such functional constraints are likely to contribute to the preservation of the *TipE *synteny block across Insecta, to maintain coordinated expression of gene family members with or without the genomic constraint of intronic nesting. Examining the insect *TipE *gene family reveals several key evolutionary events that characterize the evolution of genes within their genomic contexts: intronic nesting, escape from nesting, overlapping exons, retrotransposition, translocation, gene loss, coordinated regulation, and conserved synteny. Exploration of the evolutionary histories of genes maintained in such synteny blocks therefore offers the opportunity to identify putative structural and functional constraints on gene rearrangements and their relative contributions to genome evolution.

The common ancestry of the TipE-like and Slo-beta auxiliary subunits suggests a remarkable plasticity in the evolution of mechanisms for regulating ion channel activities. An ancestral ion channel auxiliary subunit appears to have evolved to regulate calcium-activated potassium currents in vertebrates and voltage-gated sodium currents in the invertebrate lineage. Thus, although the pore-forming alpha-subunits are generally well-conserved across metazoan species, their regulatory partners appear to exhibit more lineage-specific functions. Consequently, targeting the activity of such auxiliary subunits may prove a successful strategy for the development of insecticides that are not toxic to vertebrates, and a more detailed understanding of their molecular functions in different insect species may facilitate targeted interventions that minimise ecological knock-on effects. As well as regulating functional expression and gating kinetics of ion channels, auxiliary subunits are known to influence interactions with naturally-occurring toxins, such as scorpion venom peptides which inhibit big-conductance calcium channels [35]. Following Nature's lead could therefore offer novel prospects for the development of insect control strategies that target ion channel activities.

## Methods

### Genomic resources

Official gene sets and assembled genome sequences for 23 arthropod species were retrieved from publically available resources including AphidBase [36], BeetleBase [37], FlyBase [38], Hymenoptera Genome Database [39], SilkDB [40], VectorBase [41], and wFleaBase [42]; see Additional file 1, Table S2 for full details. The examined arthropod species include: Diptera - *Drosophila melanogaster *(Dmela) [43] and 11 other fruit flies [44]: *Drosophila sechellia *(Dsech), *Drosophila simulans *(Dsimu), *Drosophila erecta *(Derec), *Drosophila yakuba *(Dyaku), *Drosophila ananassae *(Danan), *Drosophila persimilis *(Dpers), *Drosophila pseudoobscura *(Dpseu), *Drosophila willistoni *(Dwill), *Drosophila mojavensis *(Dmoja), *Drosophila virilis *(Dviri), *Drosophila grimshawi *(Dgrim), as well as three mosquitoes: *Anopheles gambiae *(Agamb) [45], *Aedes aegypti *(Aaegy) [46], *Culex quinquefasciatus *(Cquin) [47]; Lepidoptera - the silk moth, *Bombyx mori *(Bmori) [48]; Coleoptera - the flour beetle, *Tribolium castaneum *(Tcast) [49]; Hymenoptera - the honey bee, *Apis mellifera *(Amell) [50] and the parasitoid wasp, *Nasonia vitripennis *(Nvitr) [51]; Hemiptera - the pea aphid, *Acyrthosiphon pisum *(Apisu) [52]; Phthiraptera - the human body louse, *Pediculus humanus *(Phuma) [25]; Crustacea - the water flea, *Daphnia pulex *(Dpule) [53]; and Arachnida - the deer tick, *Ixodes scapularis *(Iscap).

### Synteny block identification

The conserved genomic cluster of *TipE *gene family members was detected by employing synteny block identification procedures developed from a strategy based on orthologous gene anchors [20,54]. Initial pairwise species comparisons used single-copy orthologous genes from the OrthoDB [55,56], requiring at least two pairs of neighbouring orthologues and allowing one intervening orthologue at most. Subsequently, synteny block extension across multiple species was performed by successive projections of pairwise blocks across the species phylogeny.

### Gene model curation

Protein sequence comparisons of *TipE *gene family members and orthologues of CG18675 revealed several annotation errors including missing exons and split gene models. Gene model curation employed additional available information such as expressed sequenced tags (ESTs) and homology-based gene predictions using Fgenesh+ [57] and Wise2 [58] to confirm and correct the annotated gene models. HMM profiles of *TipE *gene family members and *CG18675 *orthologues used for gene predictions were built using HMMER [59]. The HMMs were also used to scan the proteomes of additional non-arthropod species including *Caenorhabditis elegans, Nematostella vectensis, Hydra magnipapillata, Strongylocentrotus purpuratus, Branchiostoma floridae, Danio rerio, Xenopus tropicalis, Gallus gallus, Bos taurus, Mus musculus *and *Homo sapiens*. Megablast and tBLASTn homology searches against the unassembled sequence trace archives, as well as the assembled genomes, were performed respectively to check for the presence of any additional genes potentially missed by the assembly or annotation procedures.

### Phylogenetic analyses

Multiple protein sequence alignments of *TipE *gene family members and CG18675 orthologues were built using MUSCLE [60], and confidently aligned regions were extracted with Gblocks [61]. Maximum likelihood phylogenetic trees were reconstructed using PhyML [62], with the Jones-Taylor-Thornton (JTT) substitution model, four relative substitution rate categories, estimated proportions of invariable sites and gamma distribution parameters, and 100 bootstrap samples. To support the phylogenetic analyses, conserved motif analysis of the protein sequences of *TipE *gene family members was performed using MEME [31], with the motif width from 6 to 50 amino acids and a maximum of 10 motifs.

## List of abbreviations

BK-beta: big-conductance calcium-activated potassium channel beta subunit; BK_Ca _channel: big-conductance calcium-activated potassium channel; DSC1: Drosophila sodium channel 1; EGF: epidermal growth factor; Flo-2: flotillin-2; GMC: glucose-methanol-choline; HMM: Hidden Markov Model; JTT: Jones-Taylor-Thornton; KCNMB4: calcium-activated potassium channel subunit beta-4; Na_V _channel: voltage-gated sodium channel; Para: paralytic; Slo-beta: Slowpoke-beta; Teh1-4: TipE homolog 1-4; TipE: temperature-induced paralytic E; VhaM9.7-c: Vacuolar H^+ ^ATPase subunit M9.7-c.

## Competing interests

The authors declare that they have no competing interests.

## Authors' contributions

JL, RMW and EMZ conceived and designed the study, and wrote the manuscript. JL performed the computational data analyses. All authors read and approved the final manuscript.

## Supplementary Material

Additional file 1 **Genomic resources and gene information**. Table S1: Details of the genes included in this study; Table S2: Details of the genomic resources used in this study. Click here for file

Additional file 2 **CG18675 MSA**. Multiple sequence alignment of CG18675 orthologues. Click here for file

Additional file 3 **Additional Figures**. Figure S1: Detailed map of the last exons of *CG18675 *and *TipE *in *Drosophila melanogaster*; Figure S2: Phylogenetic tree of *TipE *gene family members in six insect species and *Daphnia pulex*; Figure S3: Motif architectures of TipE-like proteins in six insect species and *Daphnia pulex*; Figure S4: Phylogenetic tree of Para orthologues in 11 insect species, *Daphnia pulex *and *Ixodes scapularis* Click here for file

Additional file 4 **Gene expression data**. High-throughput gene expression data of *D. melanogaster *genes. Click here for file
