# Supplementary material for: A remarkably stable TipE gene cluster: evolution of insect Para sodium channel auxiliary subunits
Source: BMC Evol Biol. 2011 Nov 18;11:337. doi: 10.1186/1471-2148-11-337 (PMC3240667; doi:10.1186/1471-2148-11-337)
Supplement: Additional file 3 — Additional Figures. Figure S1: Detailed map of the last exons of CG18675 and TipE in Drosophila melanogaster; Figure S2: Phylogenetic tree of TipE gene family members in six insect species and Daphnia pulex; Figure S3: Motif architectures of TipE-like proteins in six insect species and Daphnia pulex; Figure S4: Phylogenetic tree of Para orthologues in 11 insect species, Daphnia pulex and Ixodes scapularis [file 1471-2148-11-337-S3.PDF]

A remarkably stable *TipE* gene cluster: evolution of insect Para sodium channel auxiliary subunits

Jia Li[1,2], Robert M. Waterhouse[1,2], Evgeny M. Zdobnov*[,1,2,3]

Figure S1: Detailed map of the last exons of *CG18675* and *TipE* in *Drosophila melanogaster*.

The overlapping coding regions of the last exons of *CG18675* (blue) and *TipE* (red) are out of phase and encode two distinct terminal peptides of 37 (CG18675) and 20 (TipE) amino acids.

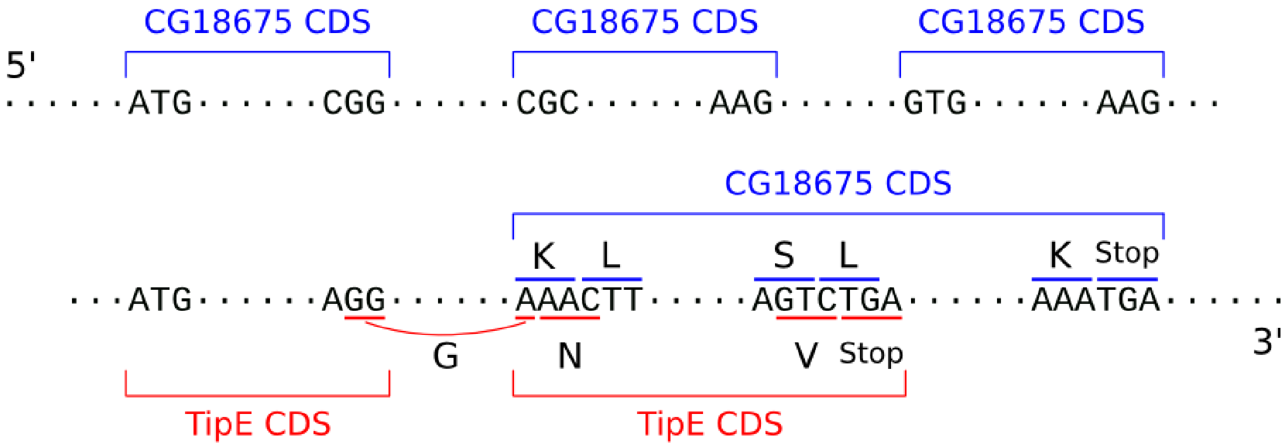

Figure S1

Figure S2: Phylogenetic tree of *TipE* gene family members in six insect species and *Daphnia pulex*.

The phylogeny of *TipE* gene family members highlights the five groups of orthologues from the crustacean water flea, *Daphnia pulex* (Dpule) and six representative insect species: the fruit fly, *Drosophila melanogaster* (Dmela); the malaria mosquito, *Anopheles gambiae* (Agamb); the silk moth, *Bombyx mori* (Bmori); the flour beetle, *Tribolium castaneum* (Tcast), the honey bee, *Apis mellifera* (Amell); and the human body louse, *Pediculus humanus* (Phuma). The clade of Teh3-Teh4 proteins is distinct from TipE-Teh1-Teh2, rooted using the distantly related vertebrate big-conductance calcium-activated potassium channel beta-4 auxiliary subunits (KCNMB4). The phylogeny resolves the *D. pulex* TipE orthologue, and although Teh1 and Teh2 orthologues are not confidently resolved, they can be identified by comparing their conserved motif architectures with the insect Teh1 and Teh2 genes (see Additional Figure 3). A single *Teh3/4*-like gene is found in the *D. pulex* genome, which probably reflects the ancestral pancrustacean genome, where a retrotransposition event before the insect radiation generated the *Teh3* retrogene. This gene duplication may have created a functional redundancy which allowed for the loss of *Teh4* orthologues in mosquitoes and the silk moth. The maximum likelihood phylogeny was built from the confidently-aligned regions of the multiple sequence alignment of member proteins (see Materials and Methods). Bootstrap support values from 100 samples are given and nodes with less than 60% support are collapsed.

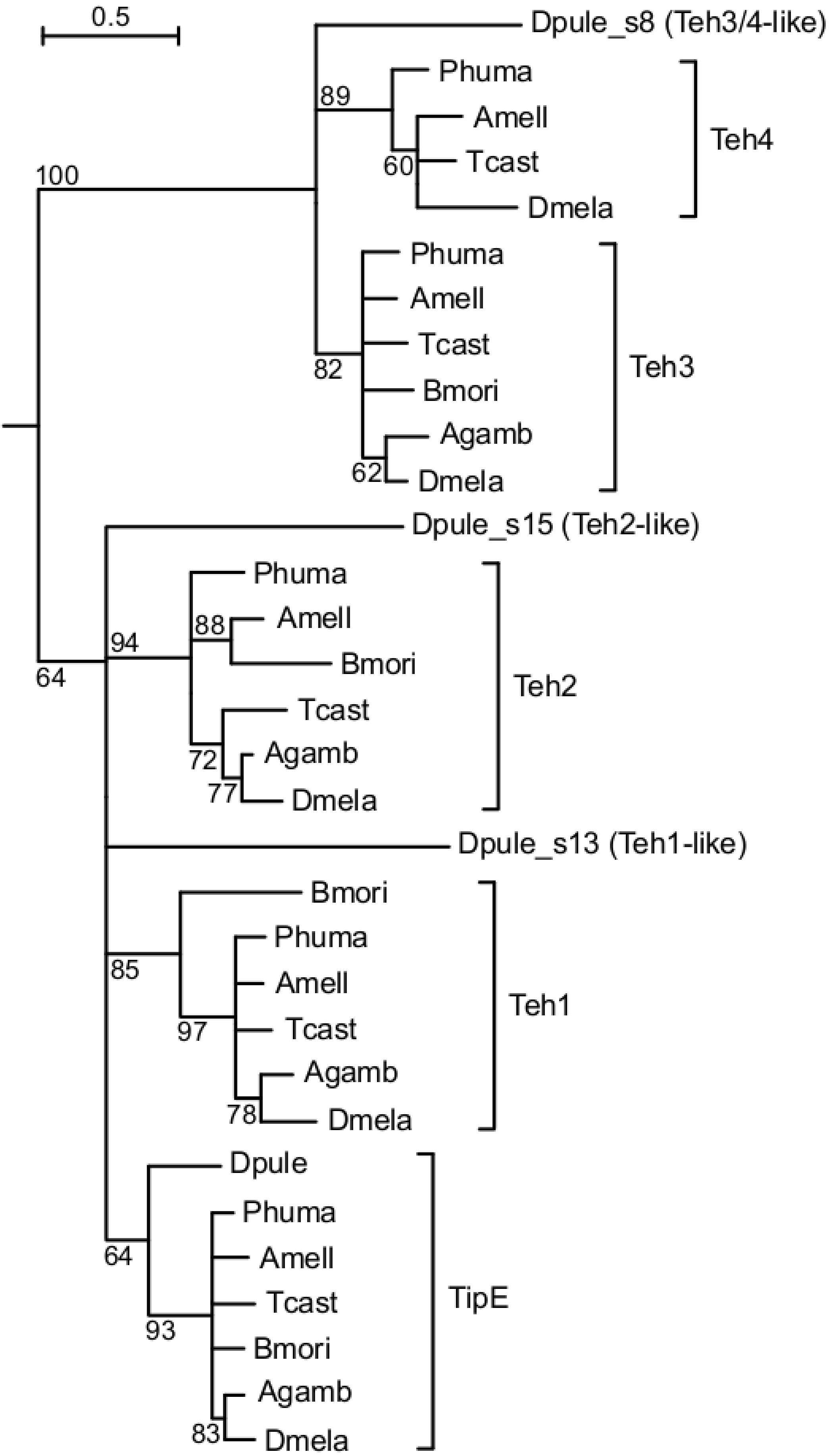
0.5
Dpule_s8 (Teh3/4-like)
89
Phuma
Amell
60
Tcast
Dmela
Teh4
100
Phuma
Amell
Tcast
82
Bmori
Teh3
Agamb
62
Dmela
Dpule_s15 (Teh2-like)
Phuma
88
Amell
94
Bmori
64
Teh2
Tcast
72
Agamb
77
Dmela
Dpule_s13 (Teh1-like)
Bmori
Phuma
85
Amell
Teh1
97
Tcast
Agamb
78
Dmela
Dpule
Phuma
64
Amell
Tcast
TipE
93
Bmori
Agamb
83
Dmela

Figure S2

Figure S3: Motif architectures of TipE-like proteins in six insect species and *Daphnia pulex.*

Conserved sequence motif analysis using MEME supports the orthology assignments, and highlights several motifs that are conserved among *TipE* gene family members:

Motif 1 contains the first transmembrane domain and the beginning part of the extracellular loop containing one conserved cysteine (Cys) that is putatively involved in disulphide bridge formation, which is found once in each gene.

Motif 2 and Motif 2' are classified as a single motif by MEME, we separated them according to the number of conserved cysteines: Motif 2 is a 3-Cys motif just after the first transmembrane domain and found once in each gene, while the third Cys is missing in Motif 2' matches and is present twice in Teh3 and Teh4 (these are not shown for TC001708 and FBgn0035504 as the p-values are below the significance cut-off, but are instead indicated by " *2' "), and the first EGF-like domain lies at the first occurrence of Motif 2'.

Motif 3 is a 2-Cys motif centred on a cysteine that is putatively involved in disulphide bridge formation, and is found once in each gene.

Motif 4 is a 1-Cys motif that also includes a cysteine that is putatively involved in disulphide bridge formation, and is found once in each gene (this is not shown for hxAUG25s13g180t1 as the p-value is below the significance cut-off, but is instead indicated by " *4 ") before the second transmembrane domain.

Motif 5 is a long 4-Cys Teh3/4-specific motif in the extended loop region, and is found in all Teh3/4 orthologues before the EGF-like domain.

Motif 6 is the second transmembrane domain found in all TipE-like proteins (this is not shown for hxAUG25s15g151t1 as the p-value is below the significance cut-off, but is instead indicated by " *6 ") except Teh1 orthologues, in which the second transmembrane domain is identified by Motif 9 instead, which is similar to Motif 6 but recognised by MEME as being distinct.

Motif 7 is a Teh3/4-specific motif in the extended loop region found in all Teh3/4 orthologues and

includes the second EGF domain.

Motif 8 is a 1-Cys motif as a subset of Motif 5 that includes a cysteine that is putatively involved in disulphide bridge formation, and is found just after Motif 2 in all of the non-Teh3/4 orthologues (this is not shown for hxAUG25s15g151t1 as the p-value is below the significance cut-off, but is instead indicated by “ *8 ”).

Motif 9 appears as a Teh1-specific motif, but as mentioned above it is similar to Motif 6 and comprises the second transmembrane domain.

Motif 10 correspond to the signal peptide, which should be present in all members, but is probably only conserved enough in Teh3/4 orthologues to be found as a motif by MEME (this is not shown for hxAUG25p1s8g331t1 as the p-value is below the significance cut-off, but is instead indicated by “ *10 ”).

The motif blocks with dashed borders are considered spurious matches to Motif 4 and Motif 2'.

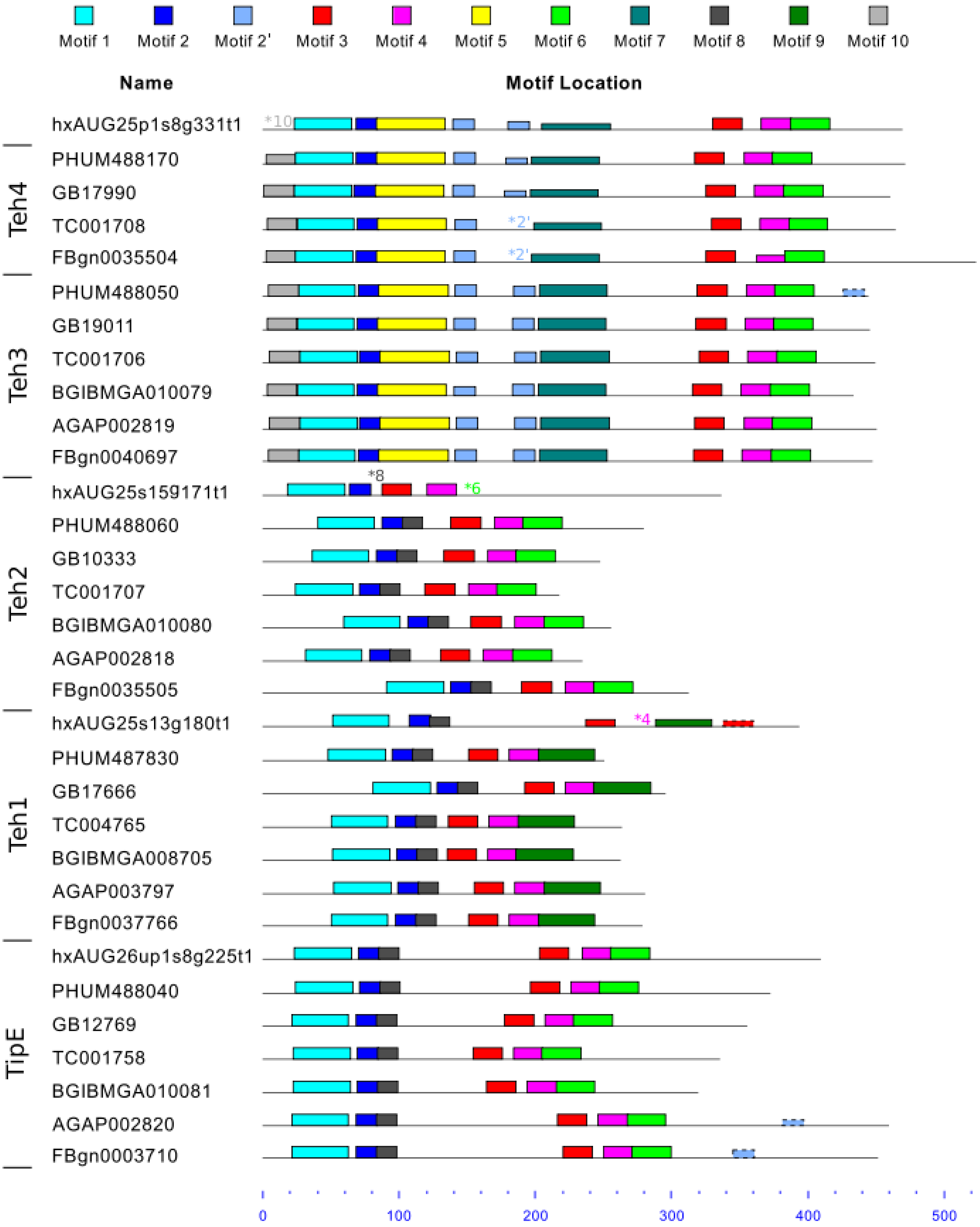
Motif 1
Motif 2
Motif 2'
Motif 3
Motif 4
Motif 5
Motif 6
Motif 7
Motif 8
Motif 9
Motif 10
Name
Motif Location
hxAUG25p1s8g331t1
PHUM488170
GB17990
TC001708
FBgn0035504
Teh4
PHUM488050
GB19011
TC001706
BGIBMGA010079
AGAP002819
FBgn0040697
Teh3
hxAUG25s159171t1
PHUM488060
GB10333
TC001707
BGIBMGA010080
AGAP002818
FBgn0035505
Teh2
hxAUG25s13g180t1
PHUM487830
GB17666
TC004765
BGIBMGA008705
AGAP003797
FBgn0037766
Teh1
hxAUG26up1s8g225t1
PHUM488040
GB12769
TC001758
BGIBMGA010081
AGAP002820
FBgn0003710
TipE
*10
*2'
*2'
*8
*6
*4
0
100
200
300
400
500

Figure S3

Figure S4: Phylogenetic tree of Para orthologues in 11 insect species, *Daphnia pulex* and *Ixodes scapularis*.

The phylogeny of *Para* gene family members confirms that orthologues of Para are present in the fruit flies: *Drosophila melanogaster* (Dmela) and *Drosophila mojavensis* (Dmoja); the mosquitoes: *Anopheles gambiae* (Agamb), *Aedes aegypti* (Aaegy), *Culex quinquefasciatus* (Cquin) – with a revised gene model; the silk moth, *Bombyx mori* (Bmori); the flour beetle, *Tribolium castaneum* (Tcast); the honey bee, *Apis mellifera* (Amell); the parasitoid wasp, *Nasonia vitripennis* (Nvitr); the pea aphid, *Acyrthosiphon pisum* (Apisu) – with a revised gene model; the human body louse, *Pediculus humanus* (Phuma); and the water flea, *Daphnia pulex* (Dpule); as well as in the closest outgroup species, the arachnid deer tick, *Ixodes scapularis* (Iscap). The maximum likelihood phylogeny was built from the confidently-aligned regions of the multiple sequence alignment of member proteins (see Materials and Methods). Bootstrap support values from 100 samples are given and nodes with less than 60% support are collapsed.

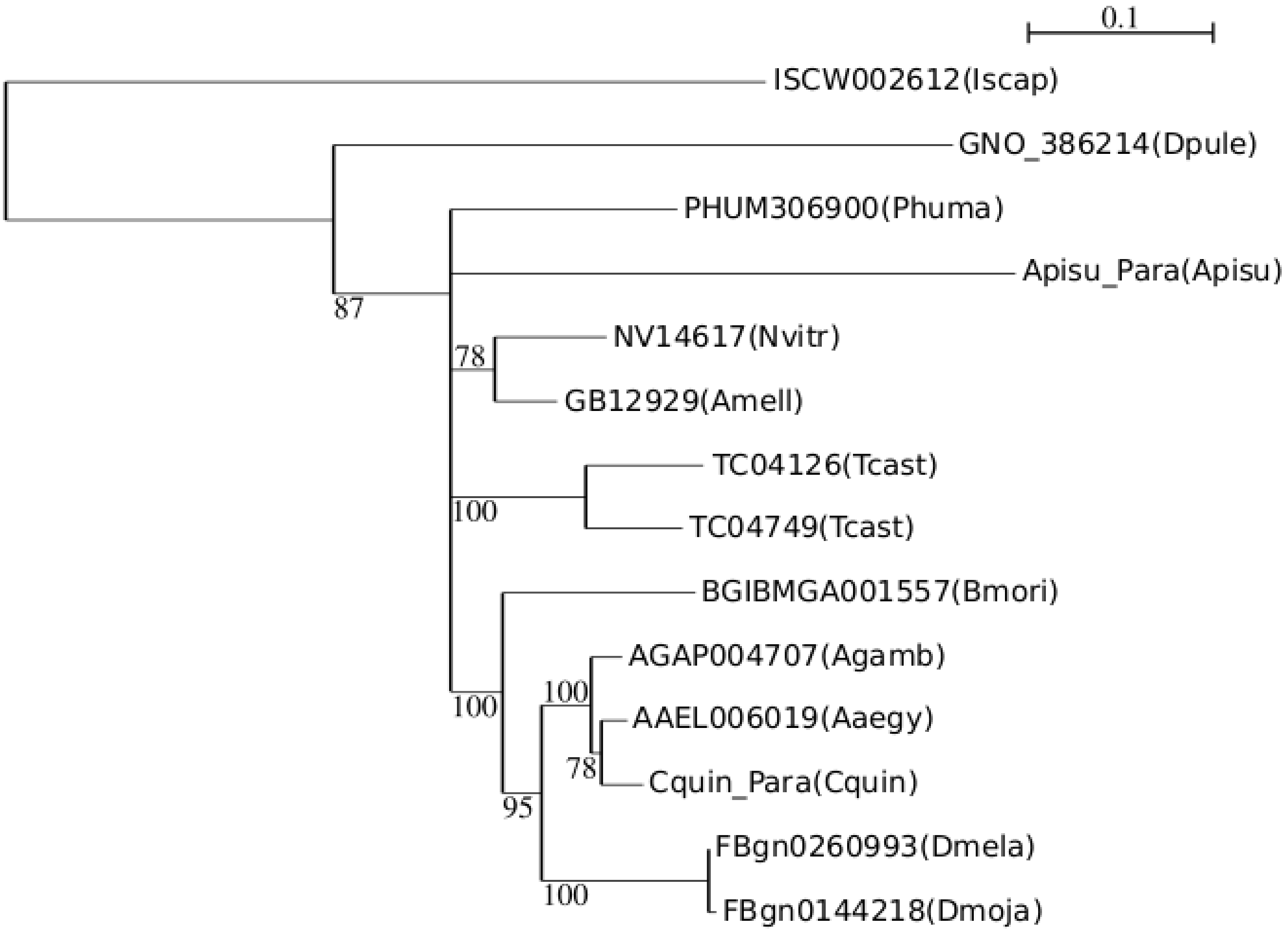
0.1
ISCW002612(Iscap)
GNO_386214(Dpule)
PHUM306900(Phuma)
Apisu_Para(Apisu)
87
NV14617(Nvitr)
78
GB12929(Amell)
TC04126(Tcast)
100
TC04749(Tcast)
BGIBMGA001557(Bmori)
AGAP004707(Agamb)
100
100
AAEL006019(Aaegy)
78
Cquin_Para(Cquin)
95
FBgn0260993(Dmela)
100
FBgn0144218(Dmoja)

Figure S4
